# Efficacy of iloprost and montelukast combination on spinal cord ischemia/reperfusion injury in a rat model

**DOI:** 10.1186/1749-8090-8-64

**Published:** 2013-04-04

**Authors:** Gokhan Lafci, Hikmet Selcuk Gedik, Kemal Korkmaz, Havva Erdem, Omer Faruk Cicek, Osman Arikan Nacar, Levent Yildirim, Ertugrul Kaya, Handan Ankarali

**Affiliations:** 1 Department of Cardiovascular Surgery, Turkiye Yuksek Ihtisas Hospital, 06100, Ankara, Turkey; 2 Department of Cardiovascular Surgery, Ankara Numune Education and Research Hospital, 06330, Ankara, Turkey; 3 Department of Histopathology, Medical Faculty, Duzce University, Konuralp, Duzce, Turkey; 4 Department of Neurosurgery, Ankara Numune Education and Research Hospital, 06330, Ankara, Turkey; 5 Department of Pharmacology, Medical Faculty, Duzce University, Konuralp, Duzce, Turkey; 6 Department of Biostatistics, Medical Faculty, Duzce University, Konuralp, Duzce, Turkey

## Abstract

**Background:**

The thoracic or thoracoabdominal aortic aneurysm surgery may cause spinal cord ischemia because of aortic cross-clamping and may result in severe postoperative complications caused by spinal cord injury. Ischemia/reperfusion injury may directly or indirectly be responsible for these complications. In this study we sought to determine whether combination of iloprost and montelukast can reduce the ischemia/reperfusion injury of spinal cord in a rat model.

**Methods:**

Medulla spinalis tissue concentrations of interleukin-6 (IL-6), myeloperoxidase (MPO) and heat shock protein 70 (HSP-70) were determined in 3 groups of Spraque Dawley rats: control group (operation with cross clamping and intraperitoneal administration of 0.9% saline, n = 7), sham group (operation without cross clamping, n = 7), and study group (operation with cross-clamping and intraperitoneal administration of iloprost (25 ng/kg) and montelukast (1 mg/kg), n = 7). The abdominal aorta was clamped for 45 minutes, with a proximal (just below the left renal artery) and a distal (just above the aortic bifurcation) clip in control and study groups. Hindlimb motor functions were evaluated at 6, 12, 24, and 48 hours using the Motor Deficit Index score. All rats were sacrificed 48 hours after the procedure and spinal cord tissue levels of myeloperoxidase, interleukin-6, and heat shock protein (HSP-70) were evaluated as markers of oxidative stress and inflammation. Histopathological analyses of spinal cord were also performed.

**Results:**

The tissue level of HSP-70 was found to be similar among the 3 groups, however, MPO was highest and IL-6 receptor level was lowest in the control group (p = 0.007 and p = 0.005; respectively). In histopathological examination, there was no significant difference among the groups with respect to the neuronal cell degeneration, edema, or inflammation, but vascular congestion was found to be significantly more prominent in the control group than in the sham or in the study group (p = 0.05). Motor deficit index scores at 24 and 48 hours after ischemia were significantly lower in the study group than in the control group.

**Conclusion:**

This study suggests that combined use of iloprost and montelukast may reduce ischemic damage in transient spinal cord ischemia and may provide better neurological outcome.

## Background

The thoracic or thoracoabdominal aortic aneurysm surgery may cause spinal cord ischemia because of aortic cross-clamping, and so severe postoperative complications may develop such as paraplegia [1,2]. The incidence of paraplegia has been reported to be as high as 35% in some series [3]. Several strategies have been applied for preventing paraplegia due to spinal cord ischemia and have included the use of shunts, systemic hypothermia, spinal fluid drainage, and ischemic preconditioning [4,5].

Previous studies showed that release of oxygen free radicals by macrophages and neutrophils are associated with ischemia/reperfusion injury (IRI) and free radical generation, lipid peroxidation or influx of calcium into cells can cause neuronal cell death in the spinal cord [6-8]. Cytokines such as interleukin-6 (IL-6) are important mediators of inflammatory response in ischemia. [9]. Myeloperoxydase (MPO) is one of the distinct indicators for the tissue infiltration of neutrophilic granulocytes and increased MPO activity as a response to the IRI is reported in the end organ tissue [10]. On the contrary, the cellular stress response can mediate cellular protection through expression of heat shock protein (HSP-70), which can interfere with the process of apoptotic cell death [11].

In literature some antioxidative and antiinflammatory agents like simvastatin [12], or N-acetylcysteine [13] are used to prevent paraplegia due to aortic ischemia in animal models. Iloprost, a stable prostacyclin analog that contributes to inhibition of platelet aggregation, vasodilatation and cytoprotection, has been considered as a neuronal cell saver and used in some experimental studies [14,15]. Montelukast, is a selective cysteinyl leukotriene (CysLT1) receptor antagonist. Since cysteinyl leukotrienes including LTC4, LTD4 and LTE4 are potent inflammatory mediators involved in the central nervous diseases such as ischemia [17], montelukast is used for the prevention of IRI in animal studies [16,17]. In this study we sought to determine whether combination of iloprost and montelukast can reduce the IRI of spinal cord in a rat model.

## Methods

### Animal preparation

This study was approved by the Animal Experimental Committee of Duzce University Graduate School of Medicine Animal care and all procedures were performed according to the guide for the care and use of laboratory animals by the National Institutes of Health.

A total of 21 male Spraque-Dawley rats weighed between 250 and 350 gr were used for the experiment. None of them had any neurological disorders before operation.

### Study groups

Twenty one rats were randomly allocated into 3 groups. In the sham group (n = 7) laparatomy was performed in the same way but without aortic occlusion. In the control group (n = 7), after laparotomy, abdominal aorta was clamped for 45 minutes with a proximal (just below the left renal artery) and a distal (just above the aortic bifurcation) clip. In the study group (n = 7) iloprost (25 ng/kg) and montelukast (1 mg/kg) were administered intraperitoneally for 30 minutes before and during the same clamping procedure as in control group. 400 IU/kg of heparin was administered intraperitoneally to all animals immediately before the procedure. All cross-clamped groups were reperfused after spinal cord ischemia of 45 minutes.

### Montelukast sodium parenteral preparation method

We did not have commercial parenteral form of montelukast. For this reason parenteral form was prepared from oral tablets. Ten tablets of montelukast 10 mg (Onceair® Merck & Co. Inc., Whitehouse Station, NJ, USA) was dissolved in 10 milliliters of ethanol. The solution was centrifuged for 5 minutes at 5000 rpm, ethanol-insoluble excipients were precipitated and the supernatant was taken. Obtained supernatant was filtered in 0.2 mM filter. The resulting solution was concentrated with evaporation method and reduced to a volume of 3 ml. In this solution, there was approximately 30 mg/mL concentration of montelukast sodium. 100 mL of this solution is injected into the high-performance liquid chromatography (HPLC) system semipreparative each time and were fractioned according to time of extracting from chromatogram.

Method which has been previously validated in HPLC device is as follows: TSP HPLC device was used [18]. Mobile phase was water-acetonitrile (5:95 vv) of 3 mL/min rate was used. Filled with C18 10x250 mm (5 mm particle diameter) RP Semipreparative (ACE) column was used. Measurings have done with 225 nm wavelength UV detector. Solutions HPLC grade were used.

Peak of montelukast sodium was at 12.115 minutes in HPLC chromatogram. Fraction was began to gather at the beginning of the peak and terminated at the end of peak. Same process was repeated until amount of montelukast sodium reached the desired level in the obtained fraction (approximately 20 again), and fractions were collected together.

The amount of drug obtained from the fraction was measured by analytical HPLC. Commercial preparation of Onceair was used as the standard for measuring. 1 tablet dissolved in 10 ml of ethanol and stock solution was obtained. Three different calibration standards established by dilution of this solution. Calibration curve was created by the ratio of the peak areas of montelukast sodium. 5 mL was injected into the fraction obtained from the analytical system. In this method, 1 mL/min rapid water-acetonitrile (5:95 vv) was used as the mobile phase. 4.6 × 250 mm C18 filled (5 mm particle diameter) RP analytical column was used (ACE). UV detector at a wavelength of 225 nm was measured.

**Figure 1:**
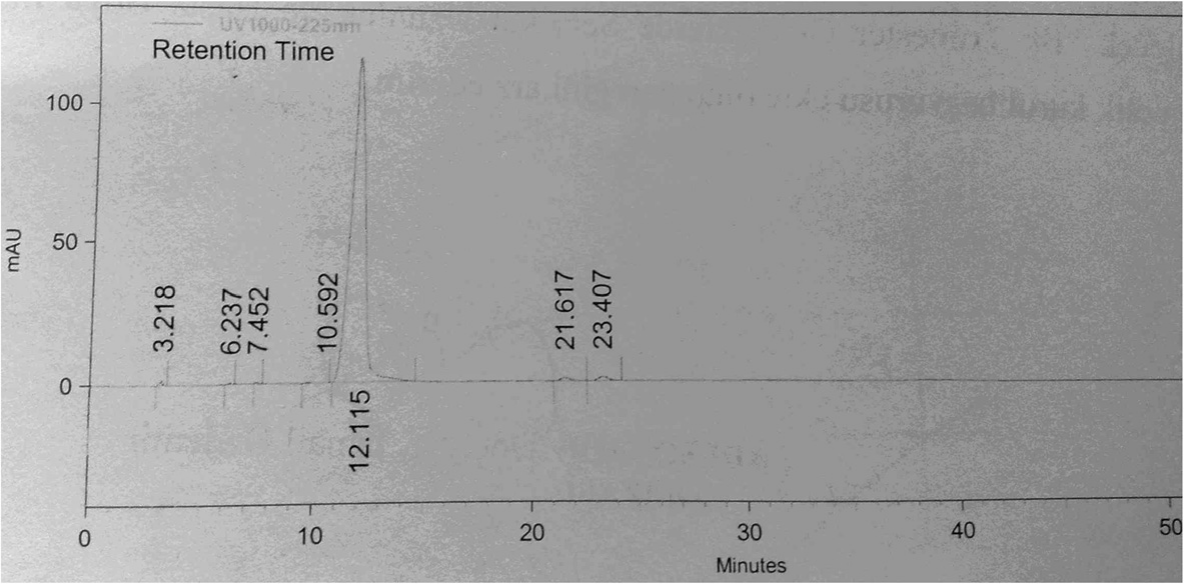
Pure montelukast sodium obtained with preparative HPLC chromatogram device.

**Figure 2:**
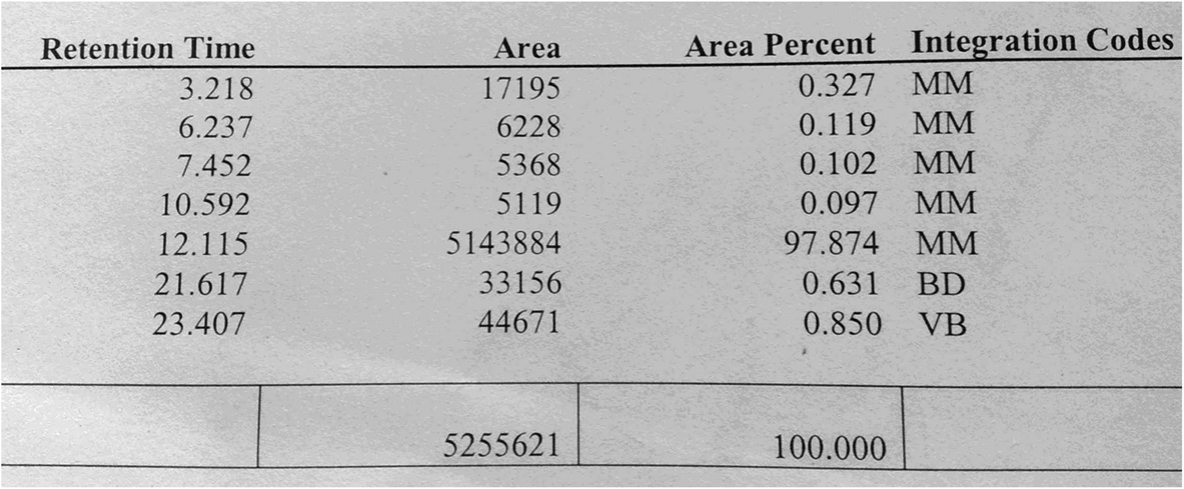
Purity ratio of montelukast sodium in HPLC.

As a result of this analysis, the purity and concentration of montelukast sodium which obtained with fraction were measured. The amount of montelukast sodium was determined by placing the peak area of obtained fraction in HPLC into calibration curve (Figure 1). Liquid phase in fraction evaporated. The remaining balance substantially diluted with distilled water to provide 10 mg/mL concentration. Purity ratio was found as 97.874%, respectively (Figure 2).

The obtained pure montelukast sodium solution was filtered with 0.2 mm membrane filter to provide sterilization, and injected rats as 1 mg/kg intraperitoneal dose (0.1 mL per kg, 0.025 mL injection, for example 250 g rat).

### Operative Procedure and Technique

Rats were premedicated with ketamine (50 mg/kg) and xylazine (5 mg/kg) intraperitoneally. The maintenance of anesthesia was established with intermittent delivery of ketamine, without endotracheal intubation and mechanical ventilation. Intraperitoneal cephalosporine (10 mg/kg) was administered before skin incision. Postoperative analgesia was provided with tramadol per 12 hours. Temperature probe was inserted into the rectum.

After the surface cleaning of the surgical area and standard midline laparatomy, the abdominal aorta was explored trough a transperitoneal approach retracting the intestines. The spinal cord ischemia was induced by clamping the aorta with mini aneurysm clamp between just below the left renal artery and just proximal to the aortic bifurcation. Temperature was maintained between 36.5 and 37.5°C during the spinal procedure with heating lamp. At the end of surgery anterior abdominal wall was sutured by using 5/0 polypropylene suture in all rats.

At 48th hour all animals were anesthetized with penthobarbital (20 mmg/kg) and sacrificed. The spinal cord tissue was extracted and fixed in buffered formalin for 7 days to analyze the tissue level of HSP-70, IL-6 and MPO and histopathological examination.

### Neurological Assessment

After reperfusion, two of the study authors who are blinded to the groups of the animals have evaluated the hindlimb motor function at 6, 12, 24, and 48 hours using the Motor Deficit Index (MDI) score, which was measured by ambulation and placing/stepping responses [19]. Walking with lower extremities was graded as follows: 0 normal, (symmetrical and coordinated ambulation); 1 toes flat under body when walking, but ataxia present; 2 knuckle walking; 3 unable to knuckle walk but some movement of the hindlimbs; and 4 no movement or drags lower extremities. A motor deficit index was calculated for each rat at each time interval.

For evaluation of sensory function, the placing/stepping reflex was assessed based on the dragging movements and responses of the hindpaw dorsum. A coordinating lifting, placing and stepping responses were observed when touching the hindpaw. Then, sensorial function was graded as follows: 0 normal; 1 some response present (weak); and 2 no response to pinch.

The maximal MDI score was 6 (score of 4 for ambulation and 2 for the placing/stepping reflex).

### Histopathological Assessment

In all animals after midline laparotomy spinal cord tissue was removed and fixed in 10% formalin for histopathological examination. Paraffin sections (4 μm thickness) were prepared. 5 μm thick sections were placed on polylysine-coated slides and stained with hematoxylin and eosin (H & E). The slides were evaluated under light microscopy (Olympus BX51; Olympus Corp., Tokyo, Japan) at 200 × magnification in terms of neuronal cell degeneration, edema, vascular congestion and inflammation. Histopathological assessment was performed by a study author, who is blinded to the groups of the animals.

### Immunohistochemical Assessment

#### Analysis of HSP

Paraffin sections (4 μm thick) were prepared. Tissue sections were deparaffinized and hydrated in xylenes and graded alcohol. The sections were incubated with primary anti-HSP70 (clone BRM.22, dilution 1/80, Biogenex, San Ramon, California) diluted in buffer. Phosphate-buffered saline (PBS) was used as negative control.

#### Analysis of IL-6

The polyclonal anti-human IL-6 receptor antibody C-20 (Santa Cruz Biotechnologies, Santa Cruz, CA, USA) was used for the immunohistochemical detection of IL-6 receptor. This antibody was diluted 1:20. IL-6 receptor immunostaining was also performed according to a streptavidin-biotin-peroxidase protocol. The secondary anti-rabbit antibody was diluted 1:500. Negative controls were performed by omitting the first antibody.

#### Analysis of MPO

The spinal cord tissue MPO activity was evaluated immuno-histochemically using an anti-MPO kit according to the manufacturer’s protocol. Briefly, samples on polylysine-coated slides were deparaffinized and rehydrated. Then, the microwave antigen retrieval procedure was performed, and the samples were incubated in a 3% H_2_O_2_ solution to inhibit endogenous peroxidase. To block nonspecific background staining, the sections were incubated with a blocking solution. Then, the sections were incubated with primary anti-MPO antibody, followed by incubation with biotinylated goat anti-mouse antibody. After incubating with the chromogenic substrate (DAB), the sections were counterstained with H & E.

The slides were examined under a light microscope. The staining of cytoplasmic MPO in the neutrophils was evaluated, and the results were expressed as the percentage of neutrophils cytoplasmically stained positive for MPO.

Tissues with no evidence of staining, or only rare scattered positive cells, less than 3%, were recorded as negative. The immunohistochemical results were evaluated for intensity and frequency of staining. The intensity of staining was graded as 0 (negative), 1 (weak), 2 (moderate), and 3 (strong). The frequency was graded from 0 to 4 by the percentage of positive cells as follows: grade 0, <3%; grade 1, 3-25%; grade 2, 25-50%; grade 3, 50-75%; grade 4, more than 75%. The index score was the product of multiplication of the intensity and frequency grades, which was then classified into a 4 point scale: index score 0 = product of 0, index score 1 = products 1 and 2, index score 2 = products 3 and 4, index score 3 = products 6 through 12.

### Statistical Analysis

Statistical analysis and calculations were performed by using SPSS 15 for Windows (Chicago,IL). Results were expressed as the mean (standard error mean). Kruskal-Wallis analysis of variance was used to detect differences among groups and statistical comparisons were made using the Mann–Whitney U test. A *p* value of ≤ .05 was considered statistically significant.

## Results

There was no statistically significant difference among the groups with respect to the body temperature. The tissue level of HSP-70 was found to be similar among the 3 groups, however, MPO and IL-6 receptor levels were lowest in the study group (p = 0.007 and p = 0.005; respectively) (Table 1).

**Table 1:** Immunohistochemical results of the medulla spinalis tissue

	**Grade**	**Control group**		**Sham group**		**Study group**		***P value***
		**Number**	**%**	**Number**	**%**	**Number**	**%**	
MPO	0	2	28.6	7	100	5	71.4	0.007
	1	5	71.4	0	0	2	28.6	
HSP	0	2	28.6	4	57.1	5	71.4	0.373
	1	4	57.1	3	42.9	2	28.6	
	2	1	14.3	0	0	0	0	
IL-6	0	0	0	5	71.4	4	57.1	0.005
	1	7	100	2	28.6	3	42.9	
	2	0	0	0	0	0	0	

**Figure 3:**
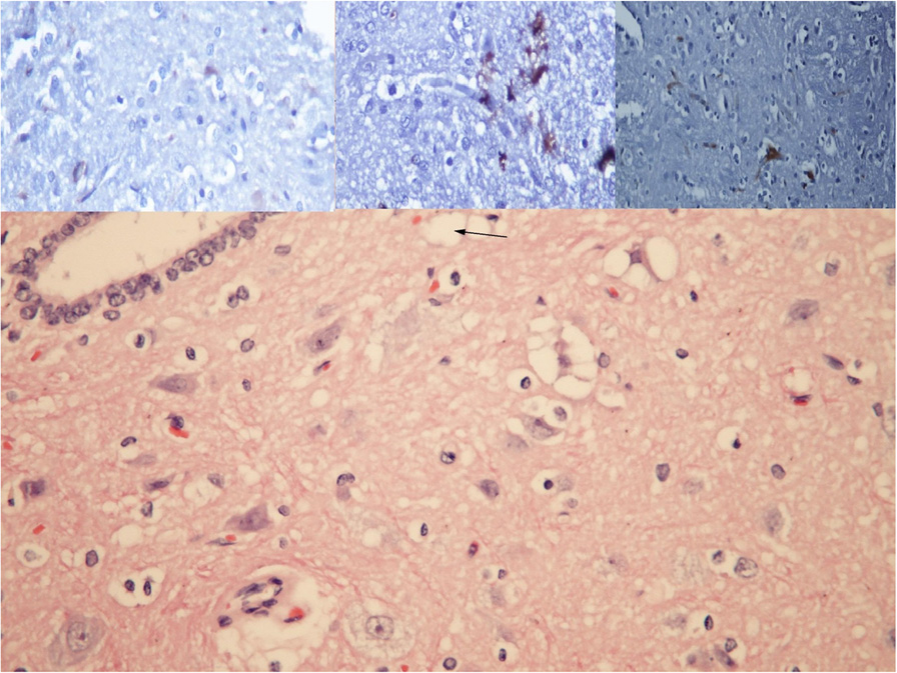
Hematoxylin and Eosin x200 staining of the medulla spinalis tissue.

In histopathologic examination, there was no significant difference among the groups with respect to the neuronal cell degeneration, edema, or inflammation, but vascular congestion was found to be significantly more prominent in the control group than in the sham or in the study group (p = 0.05) (Table 2 and Figure 3).

**Table 2:** Histopathologic results of the medulla spinalis tissue

**H&E**	**Grade**	**Control group**		**Sham group**		**Study group**		***P value***
		**Number**	**%**	**Number**	**%**	**Number**	**%**	
Edema	0	0	0	4	57.1	3	42.9	0.06
	1	6	85.7	3	42.9	4	57.1	
	2	1	14.3	0	0	0	0	
Cell Degeneration	0	1	14.3	4	57.1	3	42.9	0.289
	1	5	71.4	2	28.6	4	57.1	
	2	1	14.3	1	14.3	0	0	
Inflammation	0	3	42.9	4	57.1	4	57.1	0.304
	1	0	0	0	0	0	0	
	2	2	28.6	3	42.9	3	42.9	
	3	2	28.6	0	0	0	0	
Congestion	0	1	14.3	5	71.4	4	57.1	0.05
	1	6	85.7	2	28.6	3	42.9	

**Figure 4:**
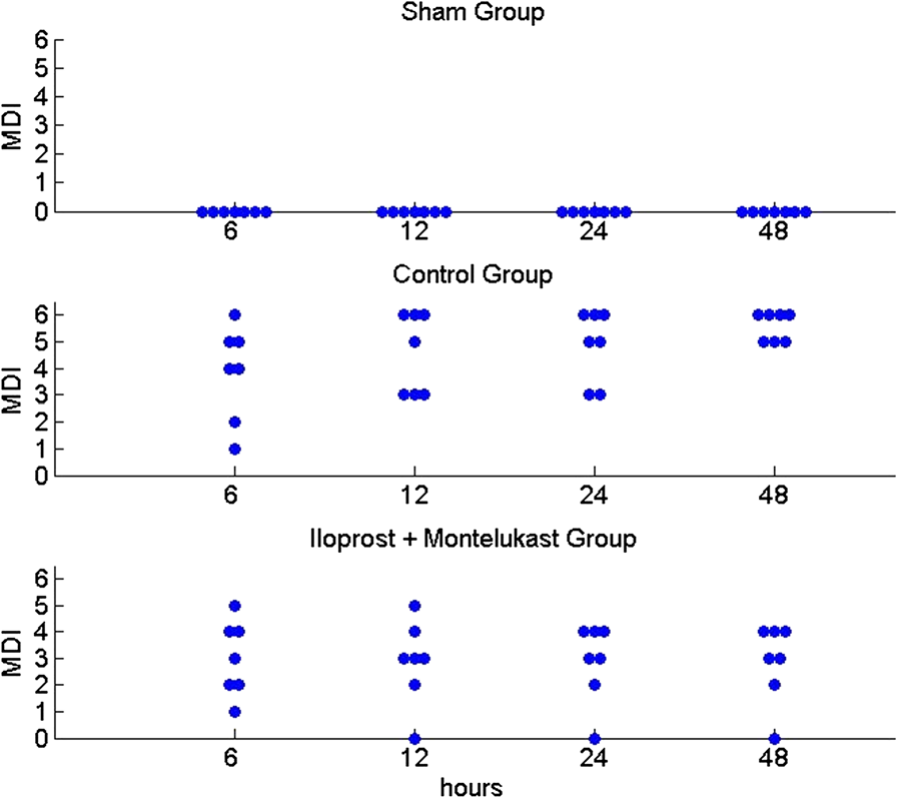
**Motor Deficit Index (MDI) assessed at 6 to 48 hours after spinal cord ischemia.** Each symbol represents the MDI score of each rat.

Ischemic injury assessed by MDI score of hindlimb was shown in Figure 4. Motor deficit index scores at 24 and 48 hours after ischemia were significantly lower in the study group than in the control group.

## Discussion

This study proposes that prophylactic administration of iloprost-montelukast combination can reduce ischemia-reperfusion injury of spinal cord in an aortic occlusion model of rats. Paraplegia is an uncommon but devastating complication, which appears after thoracic or thoracoabdominal aortic surgery due to low perfusion pressure in spite of various surgical and pharmacological interventions [20,21]. The reported incidence of paraplegia ranges from 3.8% to 17.6% [22].

Arterial vascularization of the spinal cord is similar in rat and man, with a heterosegmental aorta and few anterior radicular arteries reaching the anterior spinal artery [23]. In addition, a rat model provides important advantage for animal studies such as maintenance of normothermia throughout the operation and the development of prominent paraplegia after procedure [24]. So, many studies have been designed to detect the protective effects of different pharmacological agents during ischemia of the spinal cord in rat models as in our study.

In a previous study, Smith et al. [25] accounted for mechanism of the spinal cord IRI. They have evaluated inflammatory chemokine concentrations (interleukin-1β, IL-6, keratinocyte-derived cytokine, macrophage inflammatory protein-α, monocyte chemotactic protein-1 and TNF-α), which increased and peaked at 6 hours and 36 to 48 hours after reperfusion and they have suggested that IRI of spinal cord includes 2 phases: ischemic attack and subsequent inflammatory enhancement of the injury. Since inflammation plays a key role in spinal cord IRI, we analyzed spinal cord tissue level of IL-6 receptor, HSP70 and MPO activity to evaluate the role of iloprost-montelukast combination on the prevention of IRI. Decreased MPO activity in the study group suggests that this combination might be protective against spinal cord injury via decreased neutrophil-mediated inflammation.

In literature there are several studies that evaluating the protective effects of the different pharmacological agents and their combination (simvastatin, pentoxifylline, ilioprost and N-acetylcysteine combination, etc.) on the spinal cord IRI [12,26]. In addition to other pharmacological effects, both iloprost and montelukast are shown to be neuroprotective against IRI [16,27-29]. Montelukast, which is a CysLT1 receptor antagonist, has a dose and time dependent neuroprotective effect against IRI in a mice model and its recommended effective doses are 0.1- 1.0 mg/kg, a therapeutic window of 30 minutes even when administrated 30 min after ischemia [16,30]. Previously, Ersahin et al. [31] showed that in a rat model of spinal cord injury montelukast has neuroprotective and antiapoptotic effects on the spinal cord and it also ameliorates bladder tissue damage. They suggest that these effects are mediated by the inhibition of lipid peroxidation, neutrophil accumulation, and pro-inflammatory cytokine release. In another experimental study Genovese et al. [32] also showed that montelukast can reduce the spinal cord inflammation and tissue injury, neutrophil infiltration, TNF-α, COX-2 and pERK1/2 expression, PGE_2_ and LTB_4_ production, and apoptosis. In the present study, we sought to explore the possible additive or synergistic effect of iloprost and montelukast combination on the prevention of IRI and we found that both histopathological findings and clinical scores favor prophylactic use of these combinations in this rat model of spinal cord injury. Since two important inflammatory markers, MPO and IL-6 receptors, were found to be lower in the study group than in the control group, we propose that anti-inflammatory effects of this drug combination may reduce ischemic damage to the spinal cord. Absence of difference among 3 groups with respect to the HSP-70 level suggests that anti-apoptotic effect of this combination as a cell protective mechanism could not be concluded from this study.

Main limitation of the present study is preparation of the parenteral form of montelukast from oral tablets due to the absence of commercial parenteral form of this drug in our country.

## Conclusions

This study suggests that in rat model of spinal cord injury prophylactic use of iloprost-montelukast combination may reduce ischemic damage of spinal cord via anti-inflammatory effects and may provide better neurological outcome. Efficacy of different dosage strategies and different administration durations of these drugs should be evaluated in further studies.

## Abbreviations

IL-6: Interleukin-6; MPO: Myeloperoxidase; HSP-70: Heat shock protein 70; IRI: Ischemia reperfusion injury; CysLT1: Cysteinyl leukotriene 1; LTC4: Leukotriene C4; LTD4: Leukotriene D4; LTE4: Leukotriene E4; LTB4: Leukotriene B4; TNF-α: Tumor necrosis factor- α; COX-2: Cyclooxygenase-2; pERK1/2: Phosphorylated extracellular signal regulated kinase 1/2; PGE2: Prostaglandin E2; HPLC: High-performance liquid chromatography; H & E: Hematoxylin and eosin; MDI: Motor deficit index.

## Competing interests

The authors declare that they have no competing interests.

## Authors’ contributions

GL carried out the design and conduction of the study. HSG, KK and OFC participated in the design of the study. HE carried out the histopathological examination. OAN and LY carried out the neurological examination. EK prepared the parenteral form of montelukast. HA performed the statistical analysis. All authors read and approved the final manuscript.
